# Supplementary material for: Exploration of sensory and spinal neurons expressing gastrin-releasing peptide in itch and pain related behaviors
Source: Nat Commun. 2020 Mar 13;11:1397. doi: 10.1038/s41467-020-15230-y (PMC7070094; doi:10.1038/s41467-020-15230-y)
Supplement: Supplementary file 1 — Supplementary Information [file 41467_2020_15230_MOESM1_ESM.pdf]

Supplementary Information

Exploration of sensory and spinal neurons expressing gastrin-releasing peptide in itch and pain related behaviors

Barry et al.

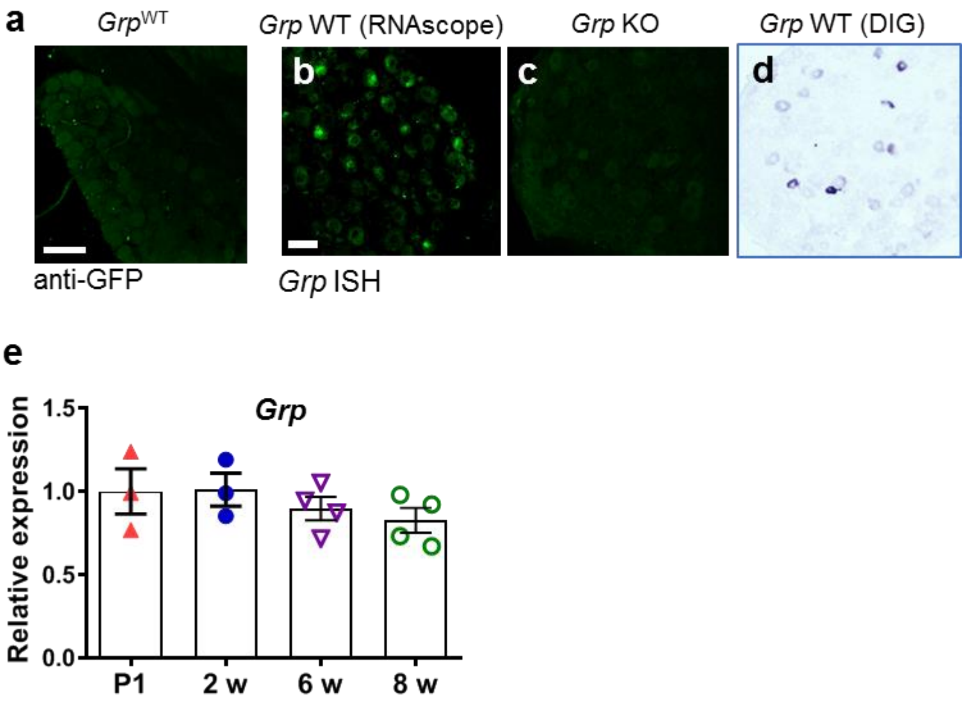

Supplementary Figure 1. Detection of *Grp* transcript in DRG. a IHC with eGFP antibody in Grp^{WT} DRG. **b** and **c** RNAScope method of *Grp* ISH in WT DRG (**b**) and *Grp* KO DRG (**c**). **d** *Grp* ISH using conventional DIG method in WT DRG. **e** The levels of *Grp* transcript in DRG are comparable at different age. P1, postnatal day 1. Data represent mean ± s.e.m., n = 3-4 mice per age group. Scale bars, 50 μm. Source data are provided as a Source Data file.

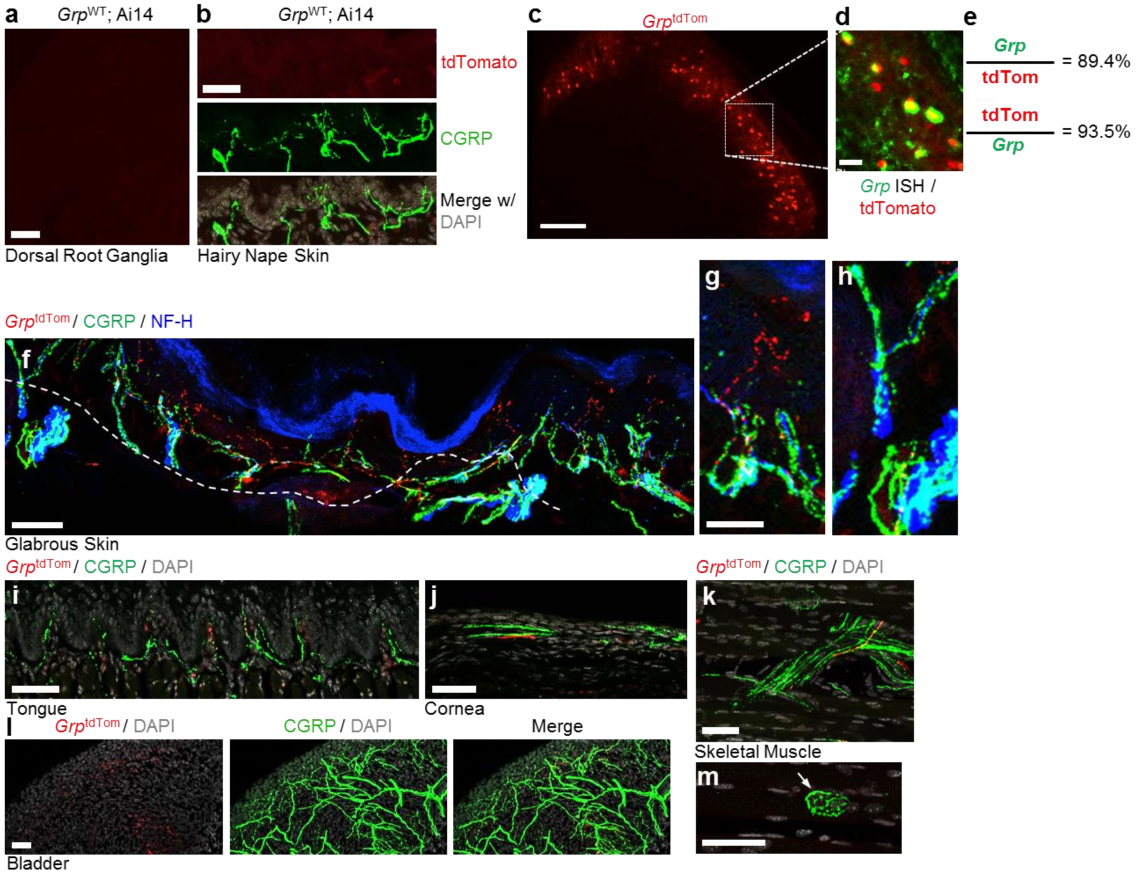

Supplementary Figure 2. Validation of *Grp*$^{\mathbf{tdTom}}$ neuorns in DRG, spinal cord and skin. a Epifluorescent image from *Grp*^{WT}; Ai14 DRG. **b** IHC images of tdTom, CGRP and DAPI in *Grp*^{WT}; Ai14 nape skin. Scale bars, 50 µm. **c** Epifluorescent images of tdTomato neurons in cervical spinal cord from *Grp*^{tdTom} mice. Scale bar, 100 µm. **d** ISH of *Grp* with tdTomato neurons. Scale bar, 20 µm. **e** Percentage of tdTom- or *Grp*-expressing neurons that express *Grp* or tdTom, respectively. n = 3 mice and 9 sections. **f-h** IHC images of tdTom, CGRP and NF-H in *Grp*^{WT}; Ai14 glabrous skin. Image in **g** shows *Grp*^{tdTom} fiber with no NF-H (blue) expression and **h** shows NF-H fibers that do not express tdTom. Scale bar in **f** 50 µm and in **g**, 20 µm. **i-m** IHC images of tdTom, CGRP and DAPI in *Grp*^{tdTom} tongue (**i**), cornea (**j**), bladder (**k**) and skeletal muscle (**l, m**). Arrow indicates neuromuscular junction in **m**. Scale bars in **l**– **m**, 50 µm.

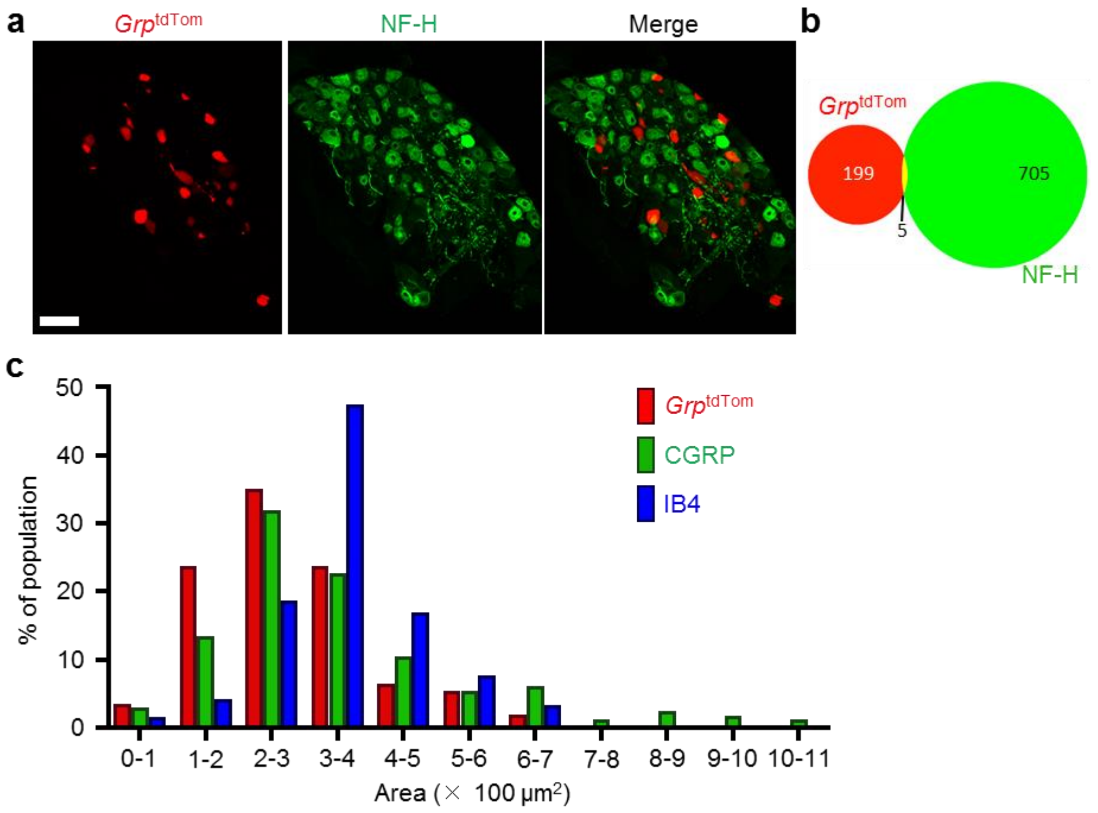

Supplementary Figure 3. Sensory *Grp*$^{\mathbf{tdTom}}$ neurons are mostly small sized neurons. **a** IHC of tdTomato and NF-H in *Grp*^{tdTom} DRG. Scale bar, 50 μm. **b** Diagram of DRG neurons with tdTomato and NF-H expression. **c** Frequency Distribution (%) of Perikarya Area (μm^2) for *Grp*^{tdTom}, CGRP or IB4 neuron populations in DRG. Neurons from 6 DRG sections were measured.

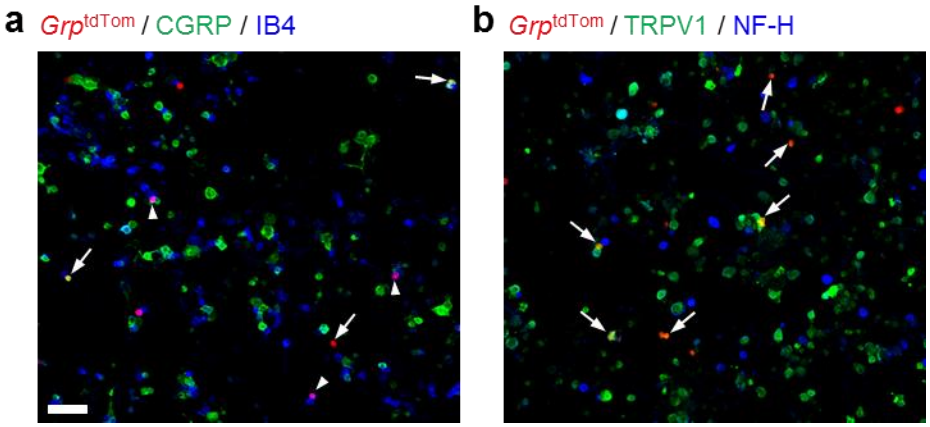

Supplementary Figure 4. Sensory *Grp*$^{\mathbf{tdTom}}$ neurons co-express CGRP and TRPV1. a IHC image of tdTomato, CGRP and IB4 from *Grp*^{tdTom} DRG cultures. Arrows indicate *Grp*^{tdTom} neurons co-expressing CGRP and arrowheads indicate IB4-binding. **b** IHC image of tdTomato, TRPV1 and NF-H from *Grp*^{tdTom} DRG cultures. Arrows indicate *Grp*^{tdTom} neurons co-expressing TRPV1. Scale bar, 100 μm.

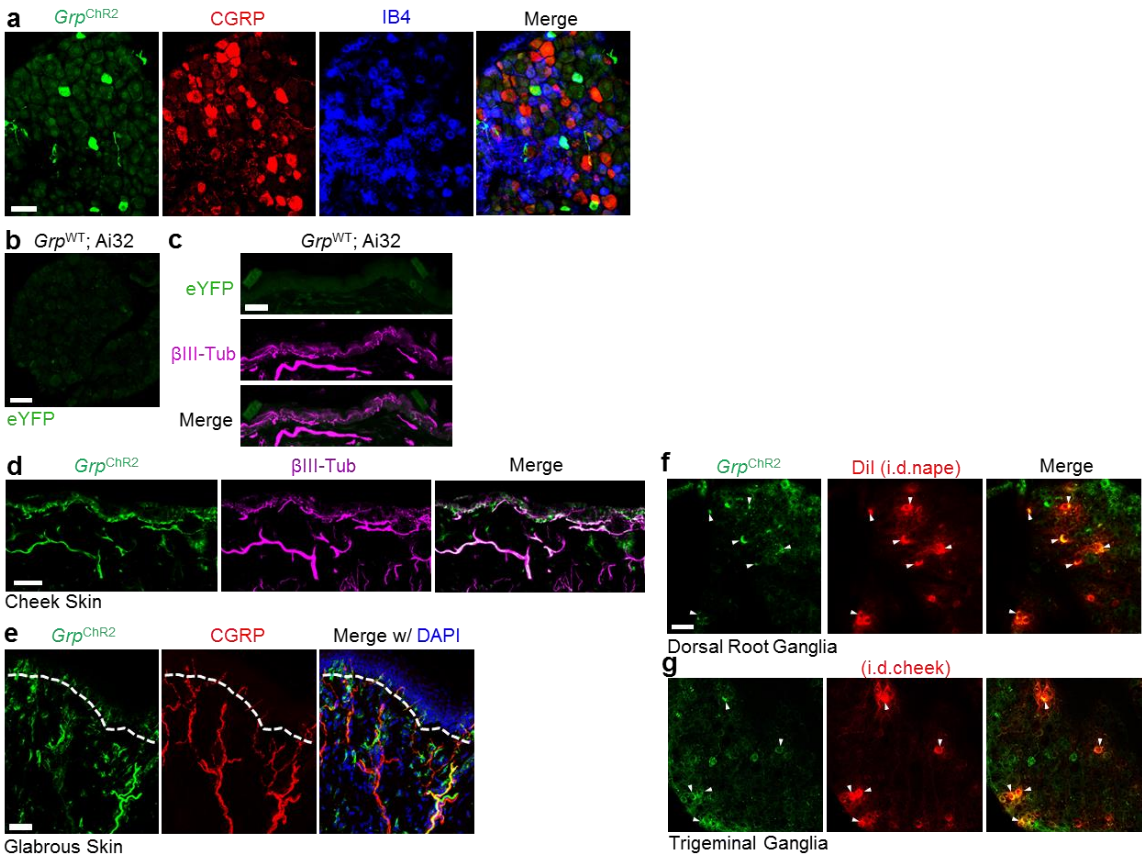

Supplementary Figure 5. Characterization of *Grp*^{ChR2} neurons in DRG and fibers in skin. a IHC images of eYFP, CGRP and IB4 in *Grp*^{ChR2} DRG. Scale bar, 50 µm. **b** eYFP image from *Grp*^{WT}; Ai32 DRG. Scale bar, 50 µm. **c** IHC image of eYFP, βIII-Tubulin and DAPI in *Grp*^{WT}; Ai32 nape skin. Scale bar, 100 µm. **d** IHC image of eYFP and βIII-Tubulin in *Grp*^{ChR2} cheek skin. Scale bar, 100 µm. **e** IHC images of eYFP, CGRP and DAPI merge in *Grp*^{ChR2} glabrous skin. Scale bar, 100 µm. **f** and **g** IHC image of eYFP and DiI in *Grp*^{ChR2} DRG (**f**) or TG (**g**) 10 days after i.d. nape or cheek injection of DiI tracer. Scale bar, 50 µm.

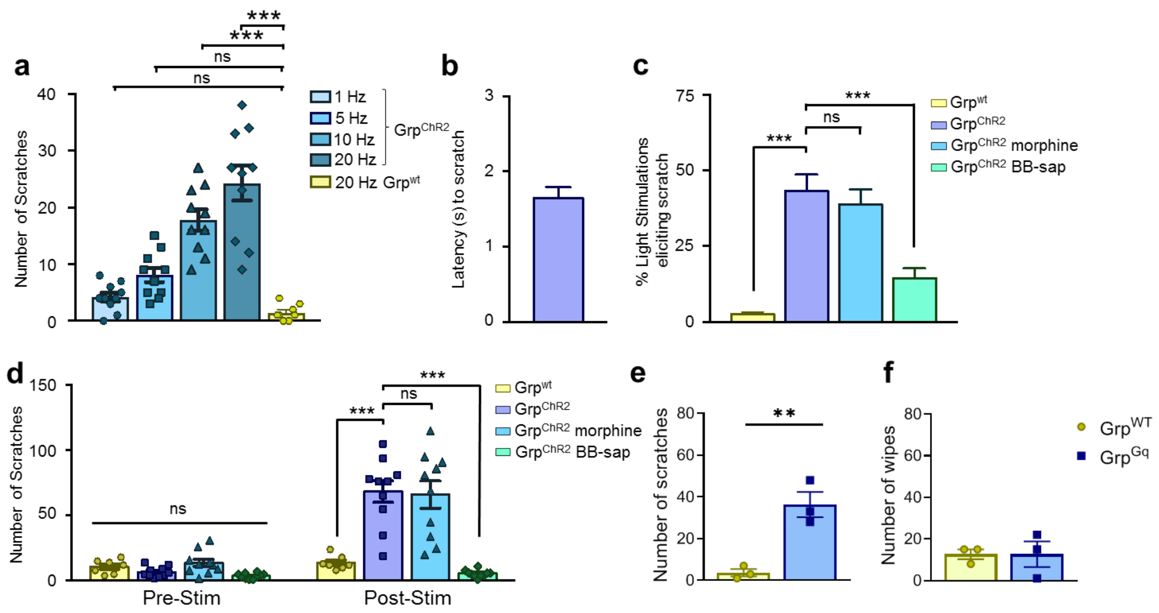

Supplementary Figure 6. Opto-stimulation of *Grp* fibers induces itch-specific behavior. a Number of scratches in 5 min induced by 3s – 1, 5, 10 or 20 Hz light stimulation of nape skin in *Grp*$^{\mathrm{ChR2}}$ and *Grp*^{WT} mice (**b**) Latency to scratch during 3s – 20 Hz light stimulation of skin in *Grp*$^{\mathrm{ChR2}}$ mice. **c** Percentage of 20 Hz light stimulations eliciting scratching behavior in *Grp*^{WT}, *Grp*$^{\mathrm{ChR2}}$, *Grp*$^{\mathrm{ChR2}}$ morphine-treated and *Grp*$^{\mathrm{ChR2}}$ BB-sap-treated mice. **d** Number of spontaneous scratches in 30 min before and after the 5-min light stimulation experiment in *Grp*^{WT}, *Grp*$^{\mathrm{ChR2}}$, *Grp*$^{\mathrm{ChR2}}$ morphine-treated and *Grp*$^{\mathrm{ChR2}}$ BB-sap treated mice. **e** and **f** Number of scratches (**e**) and wipes (**f**) in 30 min induced by CNO (1 mM) injections into the cheek skin in *Grp*^{Gq} and *Grp*^{WT} mice. Data are presented as mean ± s.e.m., $n = 8 – 10$ mice in **a-d**, $n = 3$ mice in **e** and **f**, one-way ANOVA with Tukey *post hoc* in **a** and **c**, two-way RM ANOVA with Tukey *post hoc* in **c**, unpaired *t* test in **e** and **f**, $**p < 0.01$, $***p < 0.001$, ns – not significant. Source data are provided as a Source Data file.

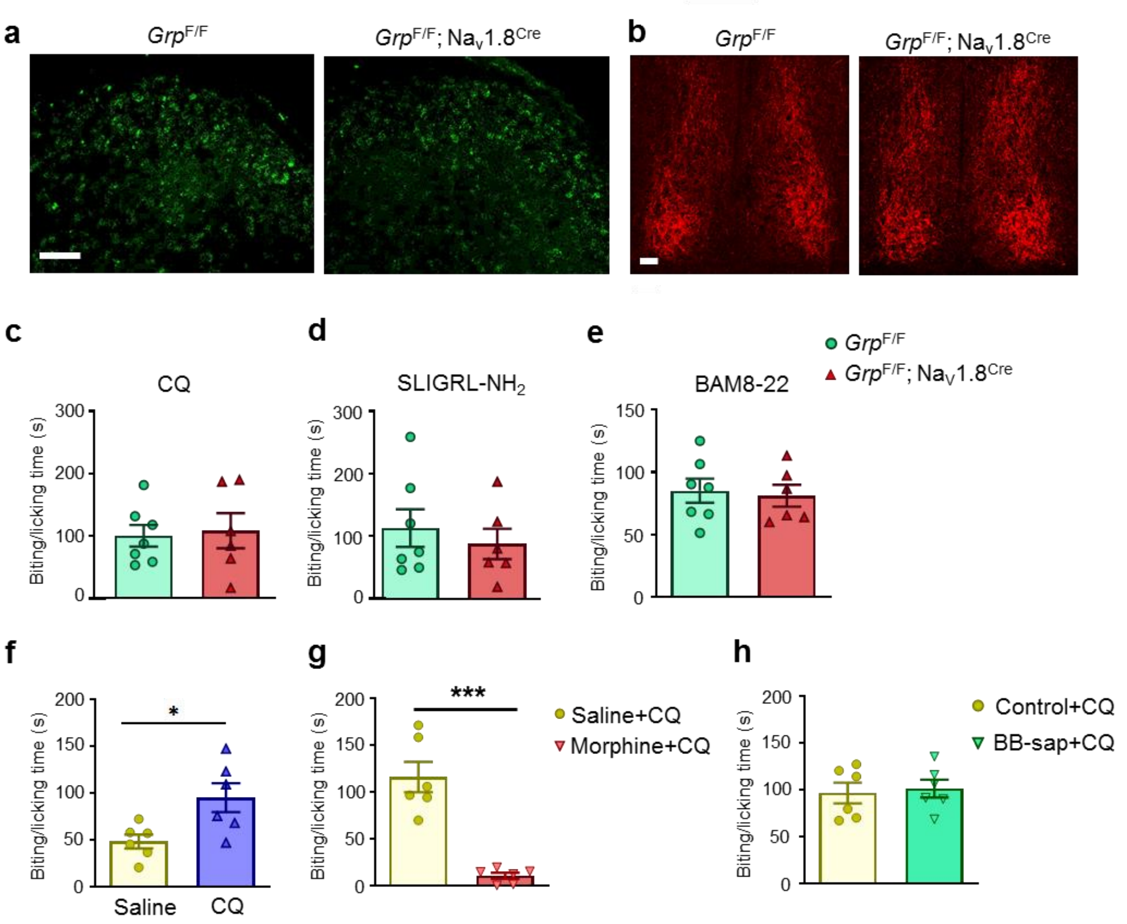

Supplementary Figure 7. Conditional deletion of sensory *Grp* is specific and does not affect pain responses. a ISH images of *Grp* expression in spinal cord sections from *Grp*$^{F/F}$ and *Grp*$^{F/F}$; Na$_V$1.8^{Cre} mice. Right panels are high power images of the boxed areas in left panels. Scale bar, 100 µm for left panels, 20 µm for right panels. **b** IHC of GRP in the suprachiasmatic nucleus from *Grp*$^{F/F}$ and *Grp*$^{F/F}$; Na$_V$1.8^{Cre} mice. Scale bar, 50 µm. **c-e** Mean biting/licking time induced by i.d. calf injection of (**c**) CQ (100 µg), (**d**) SLIGRL-NH$_2$ (100 µg), and *(***e***)* BAM8-22 (100 µg) in *Grp*$^{F/F}$ and *Grp*$^{F/F}$; Na$_V$1.8^{Cre} littermates. **f** Mean biting/licking time induced by i.d. calf injection of saline (10 µl) and CQ (100 µg). **g** Mean biting/licking time induced by i.d. calf injection of CQ (100 µg) following i.t. injection of saline (10 µl) or morphine (0.3 nmol). **h** Mean biting/licking time induced by i.d. calf injection of CQ (100 µg) on control mice and BB-sap treated mice. Data are presented as mean ± s.e.m., n = 6-7 mice. *$p < 0.05$, ***$p < 0.001$, unpaired t test. Source data are provided as a Source Data file.

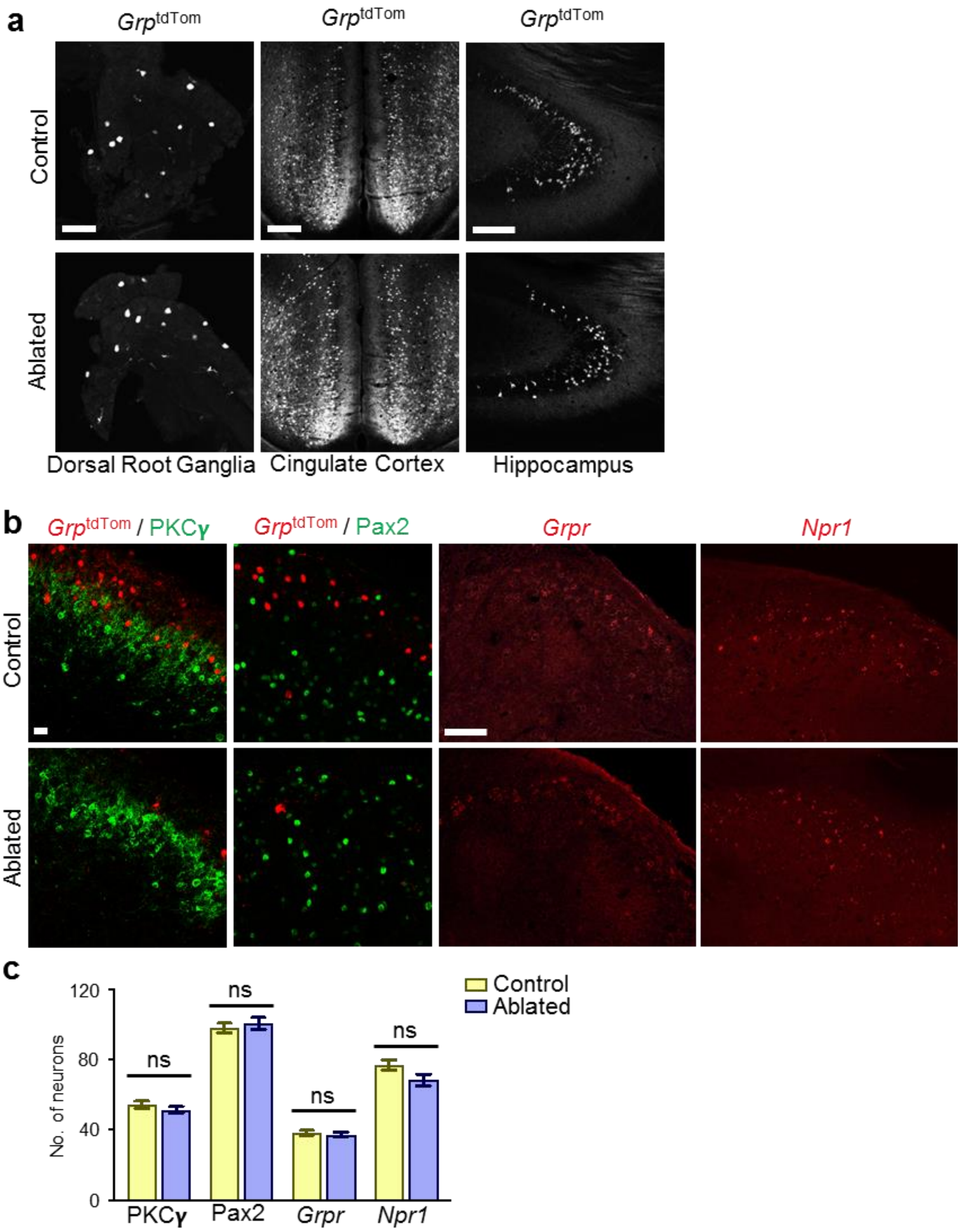

Supplementary Figure 8. Ablation of *Grp*^{tdTom} neurons is restricted to the spinal cord. **a** Epifluorescent images of tdTomato neurons in DRG, cingulate cortex and hippocampus from *Grp*^{tdTom} control and *Grp*^{tdTom} ablated mice. Scale bars, 50 μm for DRG, 100 μm for cingulate cortex and 100 μm for hippocampus, respectively. **b** IHC images of tdTom with PKCγ or Pax2 and ISH images of *Grpr* or *Npr1* expression in cervical spinal cord sections from *Grp*^{tdTom} control and ablated mice. Scale bars, 20 μm for tdTom and 100 μm for ISH. **c** Mean number of PKCγ, Pax2, *Grpr* or *Npr1* neurons from control and ablated mice. Data are presented as mean ± s.e.m., $n = 3$ mice and 10 sections, unpaired *t* test in **c**. Source data are provided as a Source Data file.

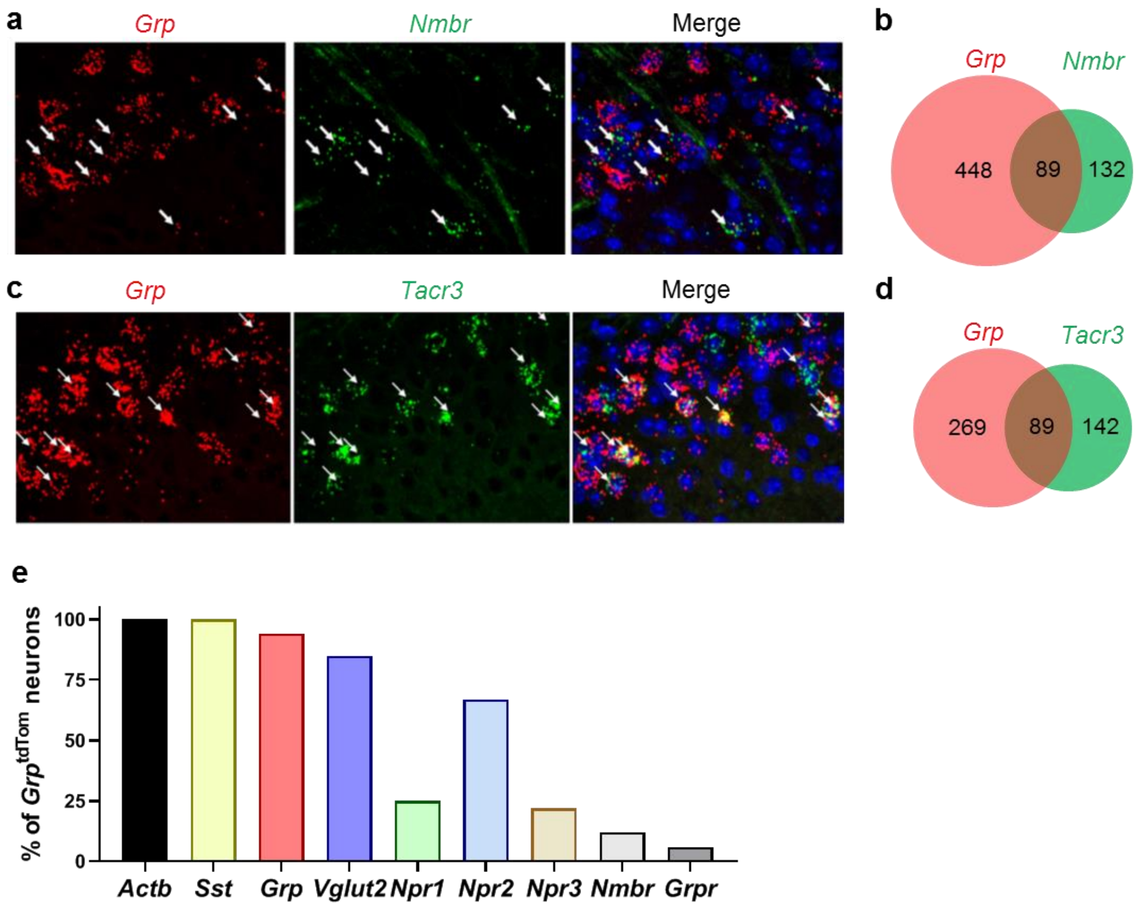

Supplementary Figure 9. Spinal GRP neurons are heterogenous. a ISH images of *Grp* and *Nmbr* in the superficial dorsal horn. **b** Diagram of overlapping expression of *Grp* and *Nmbr* in the superficial dorsal horn. **c** ISH images of *Grp* and *Tacr3* in the superficial dorsal horn. **d** Diagram of overlapping expression of *Grp* and *Tacr3* in the superficial dorsal horn. **e** Single-cell qRT-PCR results from *Grp*^{tdTom} dorsal horn neurons. n = 10-15 neurons.

Supplementary Table 1. Fiber projections in *Grp*^{tdTom} mice.

Tissue	Fibers	Tissue	Fibers
Skin (Hairy, Glabrous, Mystacial)		Retina	+(rare)
Unmyelinated:		Cornea	+(rare)
Epidermal free nerve endings	+	Tongue	+(rare)
Circular/penetrating follicle neck endings	+	Bladder	+(rare)
Bush/cluster endings	+		
		Skeletal muscle	-*
Myelinated:		Esophagus	-
Circumferential nerve endings	-	Heart	-
Lanceolate/club endings	-	Intestine	-
Meissner corpuscles	-	Kidney	-
Merkel cells and endings	-	Liver Lung	-
Sebaceous gland endings	-	Testis	-
Vibrissa follicle-sinus complex (Vib)	-	Smooth Muscle	-

* *Grp*^{tdTom} fibers were observed only in nerve bundles going through muscle but did not innervate any muscle fibers.
