# Supplementary material for: Exploration of sensory and spinal neurons expressing gastrin-releasing peptide in itch and pain related behaviors
Source: Nat Commun. 2020 Mar 13;11:1397. doi: 10.1038/s41467-020-15230-y (PMC7070094; doi:10.1038/s41467-020-15230-y)
Supplement: Supplementary file 3 — Description of Additional Supplementary Information [file 41467_2020_15230_MOESM3_ESM.pdf]

Description of Additional Supplementary Files

File Name: Supplementary Movie 1
Description: 20 Hz Light stimulation of nape skin in Grpwt control mice.

File Name: Supplementary Movie 2
Description: 20 Hz Light stimulation of nape skin in GrpChR2 mice.
