# Supplementary material for: Exploration of sensory and spinal neurons expressing gastrin-releasing peptide in itch and pain related behaviors
Source: Nat Commun. 2020 Mar 13;11:1397. doi: 10.1038/s41467-020-15230-y (PMC7070094; doi:10.1038/s41467-020-15230-y)
Supplement: Supplementary file 6 — Reporting summary [file 41467_2020_15230_MOESM6_ESM.pdf]

nature research

Corresponding author(s): NCOMMS-19-10152A

Last updated by author(s): Feb 21, 2020

Reporting Summary

Nature Research wishes to improve the reproducibility of the work that we publish. This form provides structure for consistency and transparency in reporting. For further information on Nature Research policies, see Authors & Referees and the Editorial Policy Checklist .

Statistics

For all statistical analyses, confirm that the following items are present in the figure legend, table legend, main text, or Methods section.

n/a	Confirmed	
☐	☒	The exact sample size (*n*) for each experimental group/condition, given as a discrete number and unit of measurement
☐	☒	A statement on whether measurements were taken from distinct samples or whether the same sample was measured repeatedly
☐	☒	The statistical test(s) used AND whether they are one- or two-sided<br>*Only common tests should be described solely by name; describe more complex techniques in the Methods section.*
☐	☒	A description of all covariates tested
☐	☒	A description of any assumptions or corrections, such as tests of normality and adjustment for multiple comparisons
☐	☒	A full description of the statistical parameters including central tendency (e.g. means) or other basic estimates (e.g. regression coefficient) AND variation (e.g. standard deviation) or associated estimates of uncertainty (e.g. confidence intervals)
☐	☒	For null hypothesis testing, the test statistic (e.g. *F*, *t*, *r*) with confidence intervals, effect sizes, degrees of freedom and *P* value noted<br>*Give P values as exact values whenever suitable.*
☒	☐	For Bayesian analysis, information on the choice of priors and Markov chain Monte Carlo settings
☒	☐	For hierarchical and complex designs, identification of the appropriate level for tests and full reporting of outcomes
☒	☐	Estimates of effect sizes (e.g. Cohen's *d*, Pearson's *r*), indicating how they were calculated

Our web collection on statistics for biologists contains articles on many of the points above.

Software and code

Policy information about availability of computer code

Data collection	NIS Elements Ar, version 4.10 (Nikon)
Data analysis	Prism, version 7.0d (GraphPad)

For manuscripts utilizing custom algorithms or software that are central to the research but not yet described in published literature, software must be made available to editors/reviewers. We strongly encourage code deposition in a community repository (e.g. GitHub). See the Nature Research guidelines for submitting code & software for further information.

Data

Policy information about availability of data

All manuscripts must include a data availability statement. This statement should provide the following information, where applicable:

- Accession codes, unique identifiers, or web links for publicly available datasets
- A list of figures that have associated raw data
- A description of any restrictions on data availability

The authors declare that all data supporting the findings of this study are available within the paper and its supplementary information files.

Field-specific reporting

Please select the one below that is the best fit for your research. If you are not sure, read the appropriate sections before making your selection.

☒ Life sciences ☐ Behavioural & social sciences ☐ Ecological, evolutionary & environmental sciences

For a reference copy of the document with all sections, see nature.com/documents/nr-reporting-summary-flat.pdf

Life sciences study design

All studies must disclose on these points even when the disclosure is negative.

Sample size	Sample size were based on previous experience.
Data exclusions	There were no missing data. Data outlier was not found by the definition as a data point that is located outside 1.5 times the interquartile range above the upper quartile and below the lower quartile.
Replication	All the experiments were repeated at least two times to make sure the results are repeatable.
Randomization	Animals were randomly assigned to experimental groups.
Blinding	Experimenters were blinded to group allocation during data collection and data analysis.

Reporting for specific materials, systems and methods

We require information from authors about some types of materials, experimental systems and methods used in many studies. Here, indicate whether each material, system or method listed is relevant to your study. If you are not sure if a list item applies to your research, read the appropriate section before selecting a response.

Materials & experimental systems

n/a	Involved in the study	
☐	☒	Antibodies
☒	☐	Eukaryotic cell lines
☒	☐	Palaeontology
☐	☒	Animals and other organisms
☒	☐	Human research participants
☒	☐	Clinical data

Methods

n/a	Involved in the study	
☒	☐	ChIP-seq
☒	☐	Flow cytometry
☒	☐	MRI-based neuroimaging

Antibodies

Antibodies used	chicken anti-GFP (Aves Labs, GFP-1020), rabbit anti-GRP (1:500, Immunostar, 20073), guinea pig anti-SP (1:1000, Abcam, ab10353), rabbit anti-CGRP (1:3000, MilliporeSigma, AB15360), guinea-pig anti-TRPV1 (1:800, Neuromics, GP14100), chicken anti-NF-H (1:2000, EnCor Biotechnology, CPCA-NF-H), rabbit anti-βIII-Tubulin (1:2000, Biolegend, 802001), rabbit anti-PKCgamma (1:1000, Santa Cruz Biotechnology, SC-211), rabbit anti-Pax2 (1:300, ThermoFisher Scientific, 71-6000)
Validation	Chicken anti-GFP antibody was validated by Zylka et al., 2005.<br><br>Rabbit anti-GRP antibody validated in DRGs of Grp KO mice (Sun and Chen, 2007). Immunostaining signals were absent in Grp KO tissues. Specific immunoblotting band corresponding to pro-GRP was missing in Grp KO sample.<br><br>Rabbit anti-CGRP antibody was validated by Grill et al., 1997.<br><br>Rabbit anti-Pax2 antibody was validated for immunohistochemistry on mouse spinal cord tissues by Jing Huang et al. (Nature Neuroscience, 2018) and the manufacture.<br><br>Guinea pig anti-SP antibody was validated for immunohistochemistry on mouse spinal cord tissues by Barry et al. (Molecular Pain, 2016).<br><br>Guinea pig anti-TRPV1 antibody was validated for immunohistochemistry on mouse DRG tissues by Baiou et al. (The Journal of Comparative Neurology, 2007).<br><br>Chicken anti-NF-H antibody was widely used for immunohistochemistry and validated by the manufacture.<br><br>Rabbit anti-βIII-Tubulin antibody was validated for immunohistochemistry on mouse spiral ganglion neurons by Flores-Otero et al. (The Journal of Neuroscience, 2007).<br><br>Rabbit anti-PKCgamma antibody was validated immunohistochemistry on mouse DRG tissues by Malmberg et al. (Science 1997).

Animals and other organisms

Policy information about studies involving animals; ARRIVE guidelines recommended for reporting animal research

Laboratory animals	Adult male mice between 7 and 12 weeks old were used for experiments. The mouse lines are C57Bl/6J, Ai14, Ai32, Nav1.8Cre, Lbx1flpo, Tauds-DTR, GrpCre-Kl, and Grp floxed mice.
Wild animals	The study did not involve wild animals
Field-collected samples	The study did not involve samples collected from field.
Ethics oversight	All animal studies conformed to guidelines set by the National Institutes of Health and the International Association for the Study of Pain and were reviewed and approved by the Institutional Animal Care and Use Committee (IACUC) at Washington University School of Medicine.

Note that full information on the approval of the study protocol must also be provided in the manuscript.
